# Timing by rhythms: Daily clocks and developmental rulers

**DOI:** 10.1111/dgd.12242

**Published:** 2015-11-06

**Authors:** Alexis B. Webb, Andrew C. Oates

**Affiliations:** ^1^ The Francis Crick InstituteMill Hill LaboratoryLondonUK; ^2^ University College LondonGower StreetLondonUK

**Keywords:** circadian clock, oscillators, segmentation clock, timing

## Abstract

Biological rhythms are widespread, allowing organisms to temporally organize their behavior and metabolism in advantageous ways. Such proper timing of molecular and cellular events is critical to their development and health. This is best understood in the case of the circadian clock that orchestrates the daily sleep/wake cycle of organisms. Temporal rhythms can also be used for spatial organization, if information from an oscillating system can be recorded within the tissue in a manner that leaves a permanent periodic pattern. One example of this is the “segmentation clock” used by the vertebrate embryo to rhythmically and sequentially subdivide its elongating body axis. The segmentation clock moves with the elongation of the embryo, such that its period sets the segment length as the tissue grows outward. Although the study of this system is still relatively young compared to the circadian clock, outlines of molecular, cellular, and tissue‐level regulatory mechanisms of timing have emerged. The question remains, however, is it truly a clock? Here we seek to introduce the segmentation clock to a wider audience of chronobiologists, focusing on the role and control of timing in the system. We compare and contrast the segmentation clock with the circadian clock, and propose that the segmentation clock is actually an oscillatory ruler, with a primary function to measure embryonic space.

## Embryology and types of biological timing

**Figure 1:**
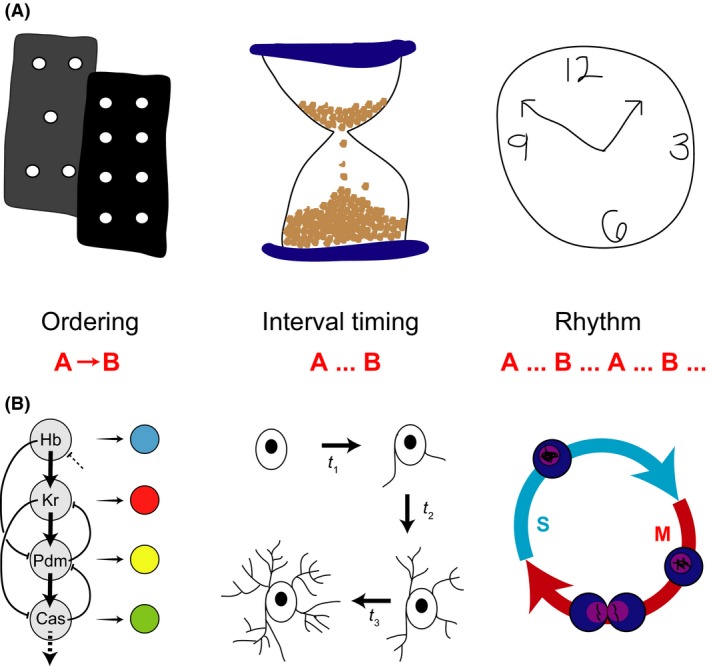
Ways of timing. (A) Schematic representations of three ways of timing biological processes. Left, ordering (cascade of dominos); Middle, interval timing (hour‐glass, or sand timer); Right rhythms (traditional mechanical clock). (B) Biological examples of timing. Left, the process of temporal patterning produces distinct sequential neural progeny from neuroblasts using a sequential cascade of transcription factors (modified from Jacob *et al*. 2004); Middle, Differentiation states of oligodendricytes occur following defined time intervals; Right, Cell cycle rhythm proceeds continuously through S to M phase and back during early rounds of cell divisions in *Xenopus* embryos.

To build a successful organism it is essential to assemble enough cells of the various differentiated types with the correct spatial arrangement within the embryo. Equally essential is the temporal coordination of proliferation, differentiation and movements of these cells during development. An embryo achieves this feat only when the right number and type of cells are in the right place at the right *time*. Despite the fundamental importance of such temporal coordination in developing tissues, its study is less mature, and perusal of a modern developmental biology textbook reveals very little relevant material. To begin, we provide a primer of three basic types of biological timing: ordering, interval timing, and rhythms, and briefly give examples of them in developmental contexts (Fig. 1). For further discussions of biological timing, see (Johnson & Day 2000) and Chapter 3 of (Phillips *et al*. 2012).

Ordering refers to the coordination of two or more sequential events in time, often with an underlying causal relationship, without explicit concern for the duration of the interval of time between them (A → B). Here, the key feature is the logical relationship between successive states. Most references to timing in developmental biology use this concept. A variant of ordering is counting, where the number of sequential events is important, but not the duration between them. For example, desert ants navigate their path by integrating distance; they do this by counting their steps (Wittlinger *et al*. 2006). Counting requires some mechanism capable of storing memory of discrete events and then acting when a particular number is passed. Interval timing concerns those processes with a well‐defined and controlled duration between two events, like the time it takes for the sand to travel through an hourglass (A… B), where the duration of the interval is important for the process. Here, the key feature is the kinetics of the process, added to its logical structure. Interval timing can become essential when parallel processes must be coordinated. While these concepts are distinct, in a temporal system in vivo, a combination may be used. For example, we suspect that many of the developmental processes currently described as ordering may well reveal properties of interval timers under further investigation.

An exemplar of ordering is the sequential production of *Drosophila* neuroblasts with different identities in ventral nerve cord, brain, and retina. A sequentially organized transcription factor network orchestrates this process, termed temporal patterning, with similar mechanisms in mammalian systems (Jacob *et al*. 2004; Li *et al*. 2003; Cepko 2014). Specific steps within these ordered cascades bear features of interval timers (Baumgardt *et al*. 2009; Kohwi & Doe 2010). A classical interval timer is the invariant timing of mouse oligodendrocyte precursor differentiation (Raff 2007). The timing between successive molts in *Drosophila* and *Caenorhabditis* ecdysis (Thummel 2001), or the duration of cell cycle phases in some contexts seem to be under mixed regulatory control: in optimal conditions they behave as genetic interval timers with well defined durations, yet these systems also operate checkpoints to ensure that conditions of resources and repair are met (Tyson & Novak 2008).

Rhythms are continuous sequences of repetitive events or state changes with regular periods, typically controlled by an oscillatory mechanism. Unlike an hourglass, a rhythm's cycle proceeds indefinitely, not having to be restarted after one interval expires (A… B… A… B…). Examples of rhythms in developmental biology are limited, but one well‐studied process is the early cleavage cell divisions of many embryos, such as *Xenopus* and *Drosophila*, where a mitotic oscillator does not pause between S and M phases (Tsai *et al*. 2014). Another developmental example is the sequential formation of metameric body segments seen in most animal phyla, such as annelids, arthropods and chordates (Couso 2009). Recent studies of a so‐called root‐tip oscillator in plants raise the possibility that the temporal control of metamerism may be a general strategy (Richmond & Oates 2012).

One way to generate a rhythm is to use a clock, which is a particular type of oscillatory system that represents time and its passage. Clocks are familiar objects to us, and we structure much of our lives and ways of thinking about time around them. However, as described above, they are only one specialized aspect of biological timing. Important for our discussion is the idea that clocks maintain a reference time that is used by the organism to organize temporal events, where the timing of the events is the critical function. We often refer to clocks as oscillators, but not all oscillators are clocks; the oscillatory mechanism used by *Dictyostelium* to generate cAMP pulses serves its key function to coordinate the cells spatially, forming spiral waves, which allows for the individual cells finding each other and thus forming a slug (Weijer 2004). The period of this oscillator changes during development. In contrast, the circadian clock is a specific biological mechanism used by an organism to temporally coordinate its internal events and external behaviors with regular periods of light and dark occurring over the continuous series of 24‐h days. The circadian clock evolved as a temporal consequence of the revolution of the Earth on its axis as it orbits the Sun.

In this review we focus on the rhythmic process of body axis segmentation seen in all vertebrate embryos giving rise to the eponymous segmented anatomical structure that defines the phylum. It is believed that a population of genetically oscillating cells termed the segmentation clock governs this process. Our purpose is to introduce readers to this developmental phenomenon, and in comparison to the circadian system, ask whether it is indeed a clock. Because of our backgrounds, our perspective emphasizes zebrafish segmentation and the central circadian pacemaker organ of mammals, the suprachiasmatic nucleus (SCN). To begin, we describe the properties of the most well defined example of clocks providing biological timing, the circadian system.

## Circadian clocks are intrinsic, daily oscillators

Our daily, 24‐h rhythm in sleep and wake is ubiquitous. Nearly every organism on Earth coordinates its activity, temperature, metabolism, and hormone release with the periods of light and darkness created by the rotation of our planet while it orbits the Sun. This intrinsic rhythm is entrained to local time through light input from specialized light‐detecting photoreceptors in the retina (Berson *et al*. 2002; Hattar *et al*. 1957; Lucas *et al*. 2007). Much like developmental biology, early data in the field relied on observation. These observations, of daily leaf movements in plants (de Mairian 2003) to eclosion of *Drosophila* larvae (Zimmerman *et al*. 1968), required a quantitative framework to describe how an oscillator could underlie this 24‐h timing (Pittendrigh 1960, 1967). So even before the first molecular components of the circadian clockwork were identified, pioneers like Colin Pittendrigh and Jurgen Aschoff developed methods to determine what could or could not be an oscillator, building a list of empirical generalizations about circadian clocks (for an excellent perspective on the history of the field, see (Roenneberg & Merrow 2005)).

**Figure 2:**
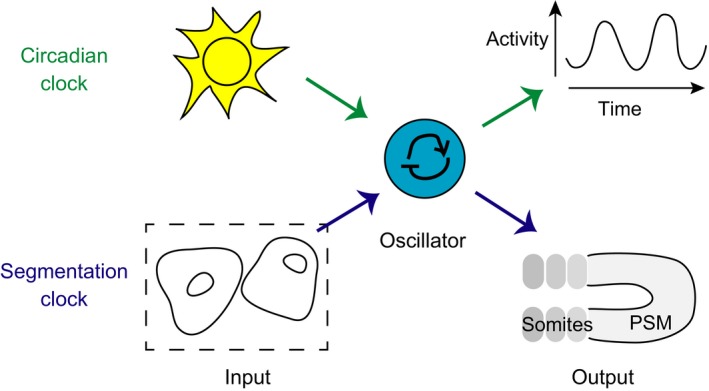
Components of rhythmic systems. We propose the following schematic representation of timing, comparing the parts of a circadian system (Eskin 1999) to the developmental process of segmentation. At the heart of each system is an oscillator. The primary input to the circadian system is light, which provides a daily, rhythmic entraining signal to the oscillator. The segmentation system has no known external rhythmic input, but cell–cell communication is a rhythmic input between cells within the tissue that leads to synchronization. The outputs of the circadian system are measured as daily rhythms in behavior and physiological processes. The segmentation system also produces a rhythmic output – the sequential formation of somites from the presomitic mesoderm (PSM).

A circadian system is often described in three parts (Eskin 1999) – an input, such as light, the oscillator itself, and an output that can be measured, like daily sleep/activity rhythms (Fig. 2). As we are discussing different ways of biological timing in this review, we will emphasize a comparison between aspects of the oscillator in the circadian system to what is known about the segmentation clock.

The first criterion for a circadian clock is that it persists and is able to generate rhythms with a period of nearly 24 h in constant conditions, i.e. without being driven by an extrinsic signal. The location of pacemaker tissues was identified in a variety of species, including birds, insects, and molluscs (Gaston & Menaker 2013; Nishiitsutsuji‐Uwo & Pittendrigh 1968; Eskin 1999). Ablation and transplant studies led to the localization of the pacemaker in mammals, the SCN in the ventral hypothalamus (Stephan & Zucker 1972; Ralph *et al*. 1990). Additionally, this circadian clock pacemaker is autonomous in various species, as its activity rhythm continues in tissue explants (Herzog *et al*. 2003; Yamaguchi *et al*. 2003), single cells (Welsh *et al*. 1995; Webb *et al*. 2009), and even in the phosphorylation of proteins *in vitro* (Nakajima *et al*. 2005).

Secondly, circadian clocks can be entrained to external signals, like light, feeding, or temperature rhythms, otherwise known as *zeitgebers* or time‐givers. Importantly, the process of entrainment is not to drive the clock, but instead provides phase cues to adjust the rhythm in time.

Finally, circadian clocks are temperature compensated, that is, they are able to maintain timing across a range of temperatures (Hastings & Sweeney 2002). The Q_10_, or temperature coefficient, is the change in the system's rate when changing the temperature by 10 degrees Celsius. In the case of a temperature‐compensated clock, the Q_10_ value is 1.

As mentioned earlier, these criteria were in place before the discovery of the so‐called “clock” genes, which we now know exist in all cells in nearly every organism. Using forward genetics, Benzer and Konopka identified the first of these in *Drosophila*. Animals with longer, shorter, and a loss of eclosion rhythms were all found to contain different mutations in the same gene, which they named *period* (Konopka & Benzer 2013). This gene was later cloned, and was the first of now dozens identified as gears within such clockworks in organisms ranging from flies to fungi to plants to mammals (Dunlap 2014). Thus, networks of transcription factors and proteins help to set the near‐24 h rhythm we observe in biological processes. The molecular era of chronobiology began with the proposal that the delay in the transcription of genes and translation of proteins, which then could return to the nucleus to inhibit the transcription of genes, would take approximately 24 h per cycle (Hardin *et al*. 2012). This idea has been refined over the last 25 years to include a whole suite of events that contribute to the daily rhythm. The field has gone on to systematically pursue understanding of how the circadian oscillator is built to maintain time. However, it is important to note that measuring and manipulating inputs to and outputs from the clock are still essential parts of research in this field.

## Somitogenesis subdivides the vertebrate embryo into segments

**Figure 3:**
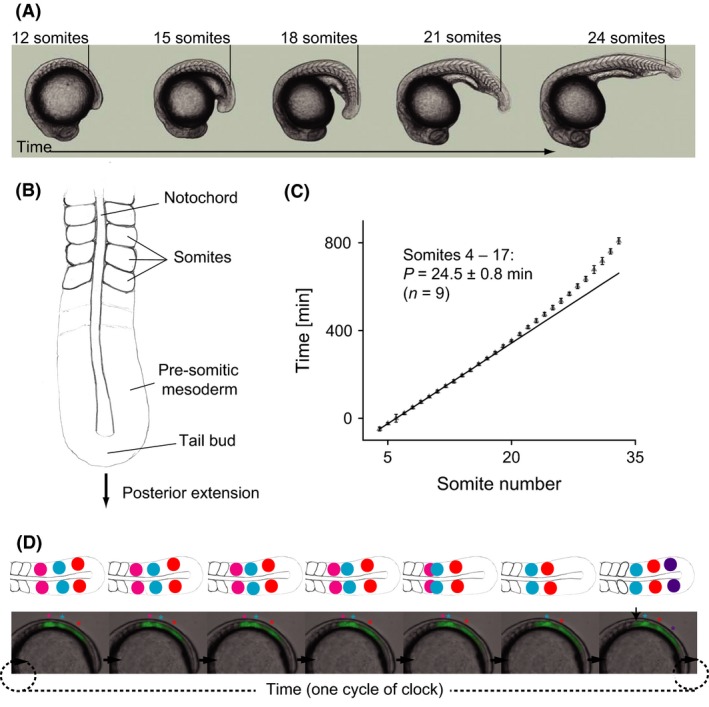
Periodic features of a developing vertebrate embryo. (A) The sequential nature of somitogenesis in a developing vertebrate embryo illustrated with a sequence of zebrafish embryos. The number and position of the most recently formed somite is indicated in each embryo. (B) Schematic diagram of the posterior end of an elongating, segmenting vertebrate embryo indicating the key structures referred to in the text. A and B (from Oates *et al*., 2002). (C) The precision of somitogenesis period demonstrated by the somite number versus elapsed time plot from a multiple‐embryo time‐lapse recording zebrafish embryogenesis (from Schröter *et al*. 2008). (D) Oscillating gene expression patterns found across the vertebrate tailbud and presomitic mesoderm (PSM) illustrated with a time series of a live reporter of the *Her1* transcription factor (green signal) in a *Looping* zebrafish embryo in lateral view (Soroldoni *et al*. 2014). Above are schematic representations of the wave pattern across the PSM from a dorsal view. Colored asterisks, corresponding to colored circles on the cartoon, indicate distinct and sequential waves of expression that move across the PSM tissue and arrest once a somite is formed (arrowhead).

The embryonic body segments of vertebrates are formed rhythmically and sequentially at the posterior end of the extending body axis (Fig. 3A). These segments are termed somites: regular‐sized, discrete blocks of cells that will later differentiate into the bones of the vertebral column and the associated muscles and skin tissue. The total number of body segments and their lengths along the axis are tightly constrained within a species, but vary widely between species with some frogs possessing as few as 10 vertebra, whereas many snakes and sharks have several hundred (Richardson *et al*. 1998). Each newly forming pair of bilaterally symmetrical somites is added to the posterior end of the row of existing somites by the morphogenetic rearrangement of a cohort of cells at the anterior end of the pre‐somitic mesoderm (PSM) (Fig. 3B). Together with the tailbud, the PSM forms the posterior‐most unsegmented tissue of the vertebrate embryo. Estimates from staged embryos indicate that the rhythm of somite formation is regular and species‐specific, ranging from approximately 30 min in zebrafish to 2 and 6 h in the mouse and human, respectively (Gomez *et al*. 2008). The striking precision of the period of this rhythm has been demonstrated by multiple‐embryo time‐lapse measurements in zebrafish (Schröter *et al*. 2008) (Fig. 3C).

With such clock‐like precision in the timing of somitogenesis, it seems plausible to expect some type of oscillatory mechanism setting the pace in the PSM and tailbud. Indeed, a molecular counterpart termed the segmentation clock and first discovered in the chick embryo (Palmeirim *et al*. 1997), underlies this morphological rhythm. Oscillating patterns of gene expression are present in the PSM and tailbud of all vertebrate embryos so far studied, suggesting the existence of a conserved genetic oscillatory mechanism (Gomez *et al*. 2008; Krol *et al*. 2004). Although species differences exist, the key features of these patterns are (i) a burst of gene expression in the tailbud and posterior PSM; (ii) the movement of a wave of gene expression anteriorly through the PSM; (iii) the slowing of the wave and its arrest in the anterior of the PSM at a position that closely prefigures prospective somites; and (iv) the repeat of the pattern with the formation of each new somite pair (Fig. 3D). The correlation of the patterns with the morphological output suggests that the segmentation clock may be directly responsible for controlling the formation of somites along the embryo's body. We next discuss a model of how a clock could be used to pattern a growing embryo.

### Somitogenesis has been described by a Clock and Wavefront model

The dominant framework for converting a temporal signal into a permanent spatial pattern in vertebrate segmentation is the Clock and Wavefront model (Cooke & Zeeman 1976; Cooke 1982). Initially motivated over 30 years ago by the desire to explain the constant size proportions of experimentally reduced *Xenopus* embryos (Cooke 1975), the key elements of the model have proved remarkably prescient. In general terms within the model, the “clock” is a cellular oscillator that ticks in each of the cells of the tailbud and PSM. These cells are synchronized by some means with their neighbors such that the entire tissue generates a coherent rhythm. The “wavefront” is a differentiation front that sweeps through the field of oscillating cells from anterior to posterior, arresting the oscillators as it passes. This front can be considered to record the phase of the oscillators such that the field of previously arrested cells shows a stable spatially periodic pattern. However, the model does not specify how this frozen pattern is subsequently converted into morphological somites.

During somitogenesis, the oscillating tissue of the PSM and tailbud is constantly regressing toward the posterior in concert with the elongation of the embryo. Supplied by proliferation and movement, cells enter the posterior of the PSM and exit at the anterior after they arrest and are incorporated into somites. Relative to this flux of cells through the PSM, the wavefront where the oscillating cells are recorded has a velocity, even though its relative position in the PSM does not change dramatically from one segment to the next.

**Figure 4:**
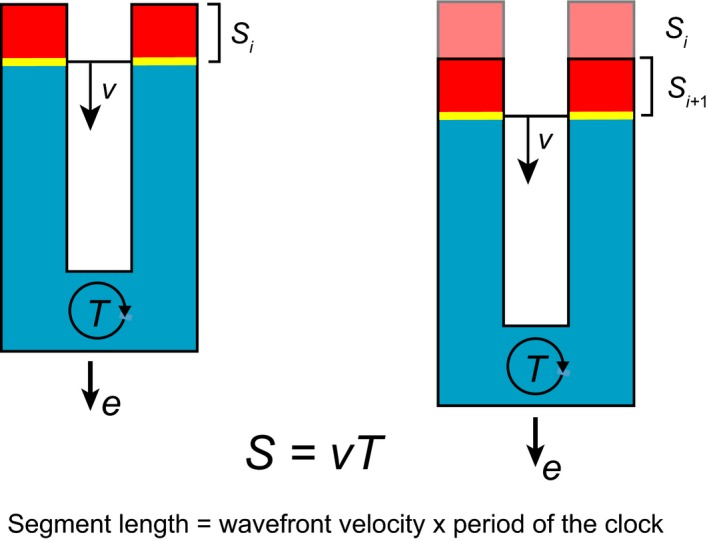
Segments as an output of a clock and wavefront mechanism. The posterior of a vertebrate embryo is illustrated in schematic form, with anterior up and posterior down. Segment length (*S*) is set by the velocity (*v*) of the wavefront (yellow) and the period of the clock (blue) (*T*). Cartoon illustrates how one segment is produced by one cycle of the segmentation clock and the velocity of the moving wavefront. For simplicity, the clock is illustrated without the characteristic waves of gene expression, as a synchronously oscillating tissue. During the cycle, the embryo has elongated with velocity *e*, due to the production of new clock cells in the posterior. If *e *= *v*, the clock tissue stays constant length, if *e *> *v* or *e *< *v*, then the clock tissue lengthens or shortens, respectively.

One prediction of the clock and wavefront model is that the segment length (*S*) is set by the period of the clock (*T*) and the velocity of the wavefront's progress across the field of cells (*v*), i.e. *S *= *vT* (Fig. 4). Another prediction is that the total number of segments in the animal's body (*n*) will be given by the duration of the segmentation process in development (*d*) and the period of the clock (*T*), i.e. *n *= *d*/*T*. These predictions are general for any similar system of oscillator and front, and do not depend on the particular molecular mechanisms at work. Thus, the important feature of this model is that it places the period of the clock in a central role in determining the anatomy of the embryo. In contrast, models, which rely on counting the number of oscillations to determine total segment number, would predict that the anatomy of the embryo would be relatively insensitive to the period of the clock. Alternative scenarios in which the “clock” plays little or no role, and instead mechanics determines the timing and length of embryonic body segments exist and have received recent experimental tests in the chick embryo (Grima & Schnell 2008; Dias *et al*. 2015).

### Segmentation period mutants

What is the role of the segmentation clock in the embryo? Might its oscillations be used to time multiple aspects of development, similar to the role of a clock in a computer, or of the circadian clock in governing behavior and metabolism? Might its cycles be counted to provide information about when to schedule other events? Or is it subservient to, or acting in parallel to, a general, perhaps global developmental timer? One way to distinguish between these models of timing, and to test the predictions of the Clock and Wavefront model, is to identify segmentation period mutants. By analogy to the circadian field, a segmentation period mutant is one where the rhythm of segmentation is altered in an otherwise normally developing embryo.

A zebrafish mutant was discovered in which somitogenesis period was reliably lengthened, but the embryo's elongation velocity, the position in the PSM where the oscillations stop, and the total duration of segmentation was not altered (Schröter & Oates 2010). In these somitogenesis period mutant embryos, an increase in segment length quantitatively matches the increase in period (*S *= *vT*), supporting the first prediction of the model. This conclusion is confirmed by recent observations of a shorter period and shorter segments, at least for the segments of the neck, in a genetically modified mouse (Harima *et al*. 1990). Furthermore, in the case of the zebrafish mutant, the total segment number is reduced both in the embryo and the adult (*n *= *d*/*T*), again in quantitative agreement with the longer period, confirming the second prediction. Combined, these simple observations suggest that the period of the segmentation clock directly controls embryonic anatomy. They also indicate that the embryo does not count the total number of segments, suggesting that the species‐specific segment number of vertebrae arises because both growth and the period of the clock are normally tightly regulated.

Regional structures such as the anus and limbs show a precise location in the body: for example, the anus of a wildtype zebrafish is always found ventral to somite 17. If the embryo does not count its total segment number, it is still possible that anatomical landmarks are positioned by counting segments. However, when the period of somitogenesis is lengthened in the mutant embryos described above, the anus is found next to segment 16, a change in position consistent with the observed increase in segment length (Schröter & Oates 2010). Thus, this and other measured anatomical and gene expression landmarks do not appear to be positioned in the body axis by counting segments. Instead, their location is determined independently of segment number, potentially using spatial cues from the size or growth rate of the embryo. These cues would constitute a different kind of time‐keeping mechanism in the embryo, perhaps akin to an interval timer. Combined, these results support a role for the period of the segmentation clock, operating under or in parallel to a more general developmental timer, in determining the body plan of vertebrates.

After 30 years, the basic predictions of the clock and wavefront model have been investigated, and seem to be still ticking. So we pose the question: is this sufficient evidence to call the timing of somitogenesis the product of a segmentation “clock” – is it an example of biological timing by rhythms?

## Comparison of circadian and segmentation clocks

**Figure 5:**
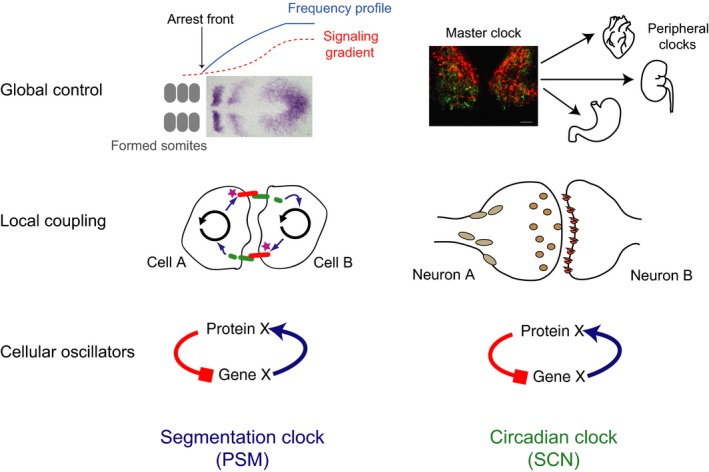
“Clocks” across scales. Current understanding of the segmentation clock mechanism (left column) has been previously described using a three‐tier model of cellular oscillators, local coupling, and global control (Oates *et al*., 2002). These tiers of organization are also applicable to the circadian clock (right). At the level of the cellular oscillators (bottom), both the segmentation and circadian clock rely on transcription‐translation feedback loops of genes and proteins. In the segmentation clock, oscillating presomitic mesoderm (PSM) cells locally couple (middle) via Delta‐Notch signaling. The mammalian circadian clock relies instead on synaptic coupling across neurons of the suprachiasmatic nucleus (SCN), other brain regions, and peripheral tissues. In addition to neurotransmitters released at synapses, neuropeptides and other humeral factors can diffuse and signal over longer distances. This long distance coupling provides global control (top) for the circadian system. The SCN acts as a master pacemaker, providing rhythmic signals to peripheral clocks to help them entrain to daily cycles, thus setting time for the entire body. In contrast, the segmentation clock does not output to other systems in the organism. Here, signaling gradients are proposed to define where in space along the PSM oscillations will arrest and a new somite will form, and provide the global control of the segmentation clock.

In this section the key functional properties of the circadian clock are used to organize a comparison with the segmentation clock (Fig. 5). We will cover the autonomous nature of pacemakers and the contribution of noisy oscillators, whether and how pacemakers from each clock entrain and synchronize. We will discuss the effects of temperature on each clock. Where possible, we briefly describe what is known about the molecular and genetic mechanisms of the segmentation clock.

### Autonomy

For a single cell to be considered a pacemaker, it must have the ability to keep time, even in the absence of inputs. Unicellular cyanobacteria (Mihalcescu *et al*. 2004), dinoflagelletes (Roenneberg & Hastings 1988), and individual retinal neurons from the sea snail *Bulla* (Michel *et al*. 1993) all can oscillate autonomously. A functional clock even exists in cyanobacteria in the absence of a cell; the phosphorylation of KaiC protein is robustly circadian *in vitro* in the presence of KaiA, KaiB, KaiC, and ATP (Nakajima *et al*. 2005). In mammals, single neurons from the SCN in the hypothalamus keep ticking with a near 24‐h period in firing rate in culture (Welsh *et al*. 1995; Herzog *et al*. 1997). Outside the SCN, many other mammalian cell types also oscillate, making organisms a veritable collection of clocks. Mammalian tissue culture cells respond to a serum shock by producing oscillations in mRNA levels of many important clock genes (Balsalobre *et al*. 1998). Later work using real‐time reporters and mathematical modeling demonstrated that mammalian fibroblasts (both NIH 3T3 and primary cells) show sustained and autonomous rhythms over a minimum of six cycles in culture, with cells having independent phases (Nagoshi *et al*. 2004; Welsh *et al*. 2004). These results prompted a closer examination of dynamic gene expression in single SCN neurons. When neurons are fully isolated from each other, they can retain the ability to oscillate, but many are sloppy and can fail to maintain rhythmicity in firing rate or clock gene expression on their own (Webb *et al*. 2009). This suggests that compared to circadian oscillators from other organisms, and fibroblasts, single SCN clocks are noisy and that coupling improves the robustness of the system (Liu *et al*. 2013; Ko *et al*. 2004; Webb *et al*. 2012). When comparing the oscillator precision of the firing activity of single SCN neurons to the SCN as a tissue, and to the behavioral activity of a mouse, the intact system is much more precise than the component pieces (Herzog *et al*. 1998).

Like the circadian system, the oscillator in single segmentation clock cells is thought to involve a negative feedback loop where the proteins of the Hes/her family of bHLH transcription factors (so‐called “cyclic genes”) feedback to repress their own transcription (Fig. 5, bottom row) (Lewis 2011). We note that unlike the distribution of near 24‐h circadian cycles, the period of the segmentation clock is much more variable across species. It is unknown whether the PSM cellular oscillator contains an additional positive feedback loop, which has been shown to play a stabilizing role in the precision of some circadian systems (Gekakis *et al*. 1968). In the circadian clock, ROR proteins activate transcription of *Bmal1*, relieving repression from REV‐ERB alpha. BMAL1 protein forms a dimer with CLOCK protein and binds to E‐boxes along the promoter elements of clock genes *Period* and *Cryptochrome*, as well as Ror (Reppert & Weaver 2002; Sato *et al*. 2004). The PSM clock also relies on hetero‐ and homo‐dimers of Hes and Her proteins to inhibit transcription (Hanisch *et al*. 2015; Schröter *et al*. 2012; Trofka *et al*. 2012). Despite the accumulated evidence, we lack a definitive test of our model for the pacemaker circuit: namely, predicting and constructing a stable oscillatory circuit with an altered period that results in a correspondingly altered body plan (Oswald & Oates 2011).

An explanted PSM can oscillate in the absence of neighboring tissues, meaning that it is, like the SCN, autonomous at the tissue level (Palmeirim *et al*. 1997). However, the question of whether individual segmentation clock cells are able to oscillate autonomously, that is, when fully separated from the tissue, has been debated for decades. Early theoretical arguments explored this possibility (Cooke & Zeeman 1976), as well as scenarios in which the oscillations were caused by interaction between the cells (Meinhardt 1986). Working from the phenotypes of mutant zebrafish and the identity of the mutated genes, cases were made both for and against cell‐autonomous oscillators (Jiang *et al*. 2008; Holley *et al*. 2002). The possibility for a negative feedback loop arising from a Her gene would be consistent with a cell‐autonomous mechanism (Hirata *et al*. 2004; Lewis 2011; Monk 2003), but the discovery of oscillations in many genes of the Wnt and FGF intercellular signaling pathways in mouse and chick raises the possibility that communication between cells may play a critical role in the generation and/or maintenance of the oscillations (Dequéant *et al*. 2012; Goldbeter & Pourquié 2008; Krol *et al*. 2004). Just as in the circadian clock, the only way to resolve this issue is to isolate cells from their neighbors and ask whether they continue with persistent oscillations.

Cells isolated from chick PSM, then dispersed and cultured at high density and fixed at subsequent time intervals show changes in cyclic gene expression (Maroto *et al*. 2005). The authors of this study were not able to distinguish between noisy autonomous oscillators and stochastic patterns of gene expression, and highlighted the need for real‐time reporters to investigate the autonomy of PSM cells. The first real‐time reporter of the segmentation clock, a luciferase reporter of Hes1 expression in mouse, allowed individual mouse PSM cells to be observed *in vitro*. Three cells dispersed in 100% serum were reported, showing four expression pulses with variable duration and amplitude (Masamizu *et al*. 2006). This study concluded that PSM cells may be “unstable” oscillators, and highlighted the role of inter‐cellular signaling in maintaining and coordinating oscillations *in vivo*. Recent work has examined isolated zebrafish segmentation clock cells using timelapse imaging, reporting multiple cycles of expression (Webb *et al*. 2014). However, cell autonomous oscillations and the role of collective processes in quality of oscillations have not been rigorously tested. Thus, the autonomy, precision, and robustness of oscillations in segmentation clock cells from all vertebrates remain open topics for investigation. We have now generated an extensive data set that provides statistical information about the period, precision and noise characteristics of single segmentation clock cells from zebrafish, suggesting that in the zebrafish, at least, they are autonomous (Webb *et al*. 2015).

### Entrainment and synchronization

The mammalian circadian clock in the SCN comprises nearly 20 000 neurons that must synchronize to each other to produce a coherent, rhythmic output to the rest of the body (Fig. 5, top right). While the cells of the segmentation clock synchronize locally via cell–cell contacts, neurons in the SCN communicate synaptically and via neuropeptides secreted over longer ranges, having axons that sometimes span the entire nucleus (Fig. 5, middle row; (Abrahamson & Moore 2001)). In addition to the entrainment of the SCN to the external environment via inputs from the retina, the neurons must entrain to each other, like the cells within the PSM, to produce a coherent output. One candidate signaling molecule for this is vasoactive intestinal polypeptide (VIP). Transgenic animals lacking VIP or its receptor, VPAC2, show disrupted behavioral rhythms (Harmar *et al*. 2013; Colwell *et al*. 2003; Hughes *et al*. 2002) and neurons from the SCN of these animals are mostly arrhythmic (Aton *et al*. 2005). Of the few remaining rhythmic cells, there is a range of intrinsic periods, which leads to desynchrony among oscillators.

We find no reported evidence of entrainment of the segmentation clock to a periodic temporal signal that is external. However, the cells of the segmentation clock are synchronized to each other locally and potentially respond globally to signaling gradients (Fig. 5, top left) (Aulehla & Pourquié 2010). Delta‐Notch coupling provides local synchronization between neighboring PSM cells that is required for proper segment formation, but not necessarily for cell autonomous oscillations to continue (Jiang *et al*. 2008; Oates & Ho 2002; Riedel‐Kruse *et al*. 2007). Delta‐Notch signaling is required for synchrony, but not necessarily rhythmic oscillations in cyclic genes to continue in the PSM tissue (Riedel‐Kruse *et al*. 2007; Delaune *et al*. 1729); however, VIP signaling may be important for imparting both synchrony to the SCN and rhythmicity to individual cells (Aton & Herzog 2005).

### Temperature compensation

A key feature of the circadian clock is the temperature compensation of the period. This is a remarkable property for a biochemical system, given that most reactions approximately double their rates for every increase in temperature of 10°C. This ensures that the period of the circadian clock is not strongly altered by fluctuations in temperature, and can therefore provide accurate timing information regardless of weather, seasons or latitude. However, daily rhythms in temperature can entrain circadian clocks, though whether these clocks are “strong” or “weak” affects the extent of their response to the temperature zeitgebers (Herzog & Huckfeldt 2002; Buhr *et al*. 2010; Abraham *et al*. 2010). Strikingly, temperature compensation of circadian cells is lost when those cells enter the mitotic program of the cell cycle (Bieler *et al*. 2014). Temperature compensation is therefore a key property of a clock, and implementing this property was one of the main technical challenges for humans in building the first reliable mechanical clocks (Sobel 1995). The chronometer invented by John Harrison in 1761 had an accuracy of about 1/5 a second per day. By improving reliability across temperatures, quartz clocks are accurate to hundredths of a second per month. In vertebrates that normally develop within narrow temperature ranges, such as birds and mammals, it is difficult to test what effect temperature has on the segmentation clock. However, in poikilotherms such as fish and amphibians where the embryos develop in environments with fluctuating temperature, it is possible to ask this question.

Imaging of zebrafish development under constant conditions has allowed for direct testing of the effects of temperature on the segmentation clock (Schröter *et al*. 2008). The overall developmental rate of the zebrafish and the rate of segmentation, proceeds faster at higher temperatures with the rate of somitogenesis showing a Q_10_ of 2.8. The length and total number of segments, however, is compensated and remains constant despite the temperature. This implies that the change in the rate of the somitogenesis is exactly matched by the change in the velocity of the wavefront and of axial extension due to temperature. How these processes are coordinated is not known. One possibility is that all the rate constants of all the reactions in the embryo are equally temperature sensitive, although this seems implausible. Alternatively, there might be a much lower number of parallel, experimentally relevant, rate‐limiting reactions that are equally temperature sensitive. Yet another scenario is that there is a hierarchy: a single rate‐limiting reaction that sets the tempo of the others.

Thus, temperature changes the period of the segmentation clock oscillator – it is not temperature compensated and in fact behaves as a reliable thermometer – placing it in contrast with the circadian clock oscillator. Instead, the property of the embryo that remains invariant under a change in temperature is the length of the segments, suggesting that the function of the segmentation clock is to measure space.

## Comparing clock centers – SCN versus PSM – spatial waves, outputs

**Figure 6:**
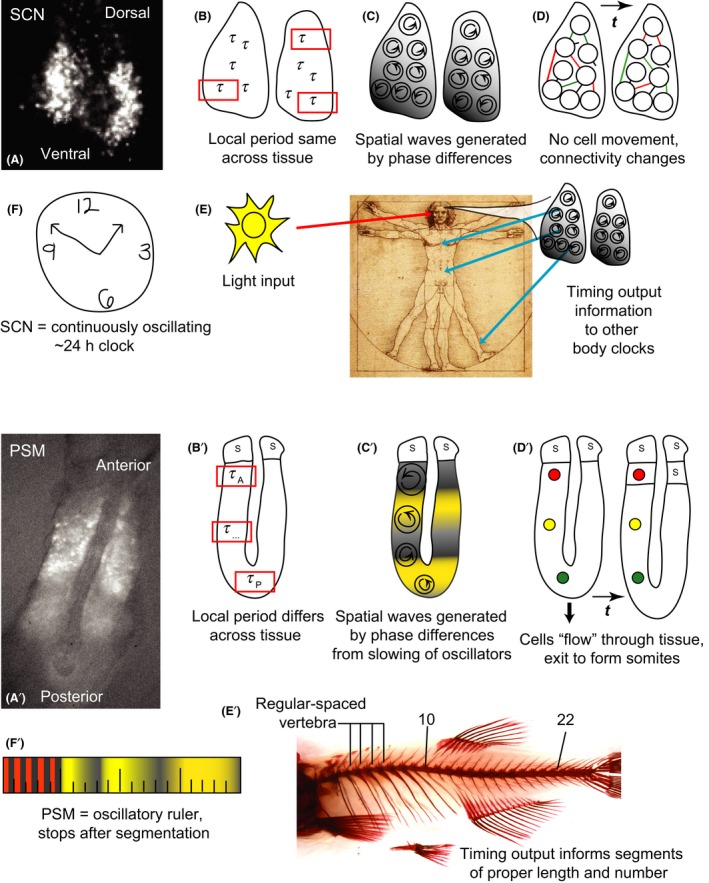
Differences between the suprachiasmatic nucleus (SCN) and presomitic mesoderm (PSM) as rhythmic centers. (Top) (A) Individual cells of the mouse SCN express *Period2*‐driven bioluminescence with waves across the tissue moving from ventral to dorsal. (B) The period of the SCN is well‐defined across the tissue: oscillations have the same period regardless of where they are observed. (C) Cells are locally synchronized, the phase waves across the tissue are generated by slight differences in phase between cells with the same period. (D) Neurons within the SCN do not move, but properties of the network connections between them can change over time. (E) *In vivo*, the SCN receives light input from the retina. Temperature cycles can also entrain the SCN. The SCN outputs crucial temporal information to other parts of the brain and body. Temporal aspects of this can change based on different inputs or network behavior within the SCN. (F) The SCN is a biological clock. (Bottom) (A') Individual cells of the zebrafish PSM express *Her1*‐driven YFP with waves of expression across the tissue from posterior to anterior. (B') The period across the PSM is not well defined, as oscillations in the reference frame of the tissue are slower in the posterior than the anterior. (C') Neighboring cells are locally synchronized, though large differences in phase exist across the tissue arising as the cells slow and eventually arrest as they form somites. (D') Cells move across the PSM while the segmentation clock ticks, generating a cellular flow in the reference frame of the tissue, and are incorporated into somites. (E') There is no evidence of external input to the PSM and the output of the segmentation clock generates a regular number and size of segments. The clock stops its output once the tissue is patterned. (F') The PSM is an oscillatory ruler.

In this section we'd like to compare the spatial organization of the two “clock” structures of the rhythmic systems we have been focusing on, the SCN and the PSM (Fig. 5, top row; Fig. 6). Both of these tissues' spatial organization is critical to their physical timing mechanisms and functions, yet they differ in subtle yet important ways that highlight the differences in function of the two systems.

Recordings of circadian clock gene driven‐bioluminescence from the SCN show waves of rhythmic gene expression that move across the tissue in subpopulations of cells (Yamaguchi *et al*. 2003; Maywood *et al*. 2006, 2013; Foley *et al*. 2015a). At first glance these patterns appear remarkably similar to the waves of gene expression observed in the PSM. SCN waves arise due to slight phase differences in the rhythms of individual cells at neighboring positions across the tissue, though the cells are thought to have identical periods. To gain some intuition about what creates such gene expression waves, one can imagine the neurons in the SCN as people in a repeating stadium wave. Every day the tissue repeats its spatial pattern of expression, and the tissue as a whole maintains a well‐defined period. This means that the period of oscillations is equivalent at every position in the tissue.

Many papers have examined inputs, light responsiveness, and rhythmic properties across the SCN, investigating what function regional differences in these elements could impart on the circadian system (Moore *et al*. 2002; Karatsoreos *et al*. 2000; Kriegsfeld *et al*. 1971; Antle & Silver 2005; VanderLeest *et al*. 2007; Meijer *et al*. 2010). Recent imaging analyses further suggest that the spatial location of a neuron within the SCN network may provide temporal information to the oscillator itself (Evans *et al*. 1979; Foley *et al*. 2015a) and to oscillators in the periphery (Evans *et al*. 2015a). This organization could also have important consequences for behavior: a recent study highlighted positive correlations between neural gene expression in sub‐regions of the SCN and the activity rhythms of mice. Interestingly, the SCN of animals that re‐entrained to new light schedules faster showed larger, late‐peaking regions of gene expression compared to mice that shifted slowly (Evans *et al*. 2011, b). Other implications for these spatial relationships and outputs are apparent in responses in aging circadian systems, as well as systems facing sleep and metabolic disorders (Ramkisoensing & Meijer 2015). Thus, because neurons in different locations of the SCN have different connections to other areas of the CNS and body, the SCN may use the spatial differences in the time at which its neurons change their circadian activity to deliver distinct temporal outputs to the different targets of the neurons.

How are these phase relationships across the SCN established and maintained? In addition to differences in inputs, it is likely that signaling between neurons plays a role. As described above, the neuropeptide VIP plays an important role in the synchrony among neurons. However, a recent study highlighted how higher concentrations of VIP could lead to differences in phase among SCN neurons and this state helped to speed re‐entrainment following a shift to a new light schedule (An *et al*. 2013). Mice that had high concentrations of VIP injected directly to their SCN re‐entrained faster following an 8‐h advance to their light‐dark schedule compared to vehicle‐injected controls. These results provocatively suggest that jetlag would be reduced by stimulating VIPergic neurons, likely by light, leading to desynchronized phasing of SCN neurons, which would allow them to shift more readily to a new entraining signal. Another player in establishing phase synchrony of SCN neurons is the neurotransmitter GABA. In the SCN, which is primarily GABAergic, the network exhibits fast excitatory and inhibitory connections, with both phasic and tonic signaling, that vary their strength by time of day (Freeman *et al*. 2011; DeWoskin *et al*. 2006). Measuring circadian gene expression under the GABA_A_ receptor blocker gabazine shows that GABA signaling injects fast time‐scale jitter into the phases of circadian neurons, desynchronizing them. Balance between slow and faster network signaling through GABA may provide a way to modulate phase for daily and seasonal changes. Therefore, not only are the inputs to the SCN network dynamic, but varied signaling amongst connected oscillators is also utilized to generate different timings between them.

In the segmentation clock, real‐time reporters have also been used to visualize the waves of gene expression across the PSM from the posterior to the anterior (Masamizu *et al*. 2006; Aulehla *et al*. 2008; Takashima *et al*. 2011; Delaune *et al*. 1729; Soroldoni *et al*. 2014). The appearance of moving waves that stop before each segment forms is not fundamentally due to movement of cells, but much like the SCN arises from a difference in the phases of neighboring oscillators across the tissue. Nevertheless, there are two important differences in the organization of the oscillators in the PSM compared to the SCN. Firstly, as mentioned in a previous section, oscillating cells enter the PSM at the posterior from the tailbud, and are removed from the anterior as each somite forms. This means that in the reference frame of the tissue, there is a continual flow of cells in the same direction as the waves of gene expression, though their movement is several times slower than the gene expression waves. In contrast, cells neither enter nor leave the SCN under normal physiological conditions in a way that generates a net flow across the tissue. Secondly, the phase differences between neighboring oscillators arise because, as they are displaced through the PSM, cells slow their oscillations gradually (Morelli *et al*. 2009; Shih *et al*. 2015). How this slowing is brought about is not yet clear, but it may be regulated by signaling gradients that span the tissue (Aulehla & Pourquié 2010). The oscillations arrest as cells are ejected from the tissue in somites. Thus, the final wave pattern that is observed across the tissue is primarily due to the differences in phase between neighboring slowing cells, with a small contribution from the transport of the cells through the tissue.

Such a complex organization of oscillations in the PSM provokes the question whether the tissue has a well‐defined period. To discuss this, it is necessary to consider both the reference frame of an individual cell, where the observer moves with the cell, and the reference frame of the tissue, through which individual cells move. Cells in the posterior that have not begun to slow oscillate with a short period and the local tissue in the posterior also has this period, in much the same way as the period of individual SCN cells matches the period of the entire SCN. This region of the tissue could be thought of as a pacemaker. Yet, by the time cells reach the anterior, their period has lengthened by two‐fold or more (Shih *et al*. 2015). Nevertheless, the period with which the tissue oscillates in the anterior is not the same as that with which individual cells oscillate (Morelli *et al*. 2009). This paradoxical statement can be resolved by imagining observing the anterior PSM through a small window, in the reference frame of the tissue, and blurring focus such that individual cells are not perceived. What is perceived is an oscillatory signal that comes from the oscillations of the individual cells convolved with their flow through the window. The period of this signal will be shorter than that observed by following the individual cells. Indeed, if the wave pattern (slowing of cells), the velocity of the flow of cells, and the position of the window (the length of the tissue) are not altered during the time of observation, the period of the oscillations in the window is the same as that of the posterior and of that in any fixed position in the tissue (Morelli *et al*. 2009). This simple observational scenario shows that the period of the segmentation clock as a whole can be well‐defined, despite the complicated organization of the oscillators. What is the situation *in vivo*; are these conditions for a well‐defined period met?

Soroldoni and colleagues examined the wave pattern produced across the PSM in zebrafish over a developmental interval including most of the trunk and part of the tail, and compared this to the timing of the formation of 15 somites during the same interval (Soroldoni *et al*. 2014). The period of oscillation in the anterior tissue reference frame, as described in the previous section, matches that of somite formation. That is, the arrival of a wave corresponds to the formation of a new somite, as expected. Surprisingly, the period of the arrival of waves in the anterior is shorter than the departure of waves in the posterior. Thus, the period of the segmentation clock is not well defined; it depends where in the tissue it is measured. Somites are forming faster than the genetic oscillations are ticking in the posterior “pacemaker” of the tissue.

In order to explain this striking phenomenon, it is necessary to recognize that the tissue length of the PSM is shortening continuously during development: the anterior of the tissue moves towards the waves that are emerging from the posterior. This is analogous to an observer moving towards a source of sound: as the observer approaches to source, the sound has a higher pitch than if the observer was at rest, a situation that is well known as the Doppler effect. Similarly, the period measured at the anterior end of the PSM is shortened, relative to the posterior, because of a Doppler effect. The Doppler effect also has the consequence that the number of waves in the tissue continuously decreases, which together with a change in the wavelength of the remaining wave pattern in the PSM, means that the wave pattern does not repeat itself exactly during the formation of each segment. Neither a change in the length of the tissue nor the change in the wave pattern with a time scale comparable to the oscillations are observed in the SCN. Although comparable measurements have not yet been made on other vertebrate embryos, these findings from the zebrafish “segmentation clock” reveal some distinctly un‐clock‐like behavior.

## Conclusions and outlook

Circadian clocks provide fitness for an organism throughout its lifetime – daily rhythms in essential biological processes, adaptability to changing seasons and reproductive cycles, and in a global, 24‐h culture, the ability to overcome challenges like resetting following trans‐meridian travel. But even beyond such modern transport, these clocks are essential for organisms getting around on Earth. For example, butterfly migration relies on continuous consultation of the insect's circadian clock in sun‐compass migration (Guerra & Reppert 2007). This example strikingly illustrates that the passing of time is read from the circadian clock. The segmentation clock is also periodic, but it exists in a single irreversible context – the one‐time patterning of the developing embryo. The segmentation clock produces an output that *is* the organism, instead of outputs used *by* the organism. The vertebrate axis is under selection for locomotion, and here it is the spatial dimension of the output that matters. The timing of the generation of segments is invisible to selection, because it occurs before locomotion, but their resulting lengths are not. Given the differences between the segmentation clock and the circadian clock we have discussed here, we are tempted to argue that the segmentation clock is not a clock at all. Its primary function is not to keep time for the developing embryo, but to measure out space. The segmentation clock is an oscillatory ruler.
